# Factors for improving reproductive performance of sows and herd productivity in commercial breeding herds

**DOI:** 10.1186/s40813-016-0049-7

**Published:** 2017-01-09

**Authors:** Yuzo Koketsu, Satomi Tani, Ryosuke Iida

**Affiliations:** School of Agriculture, Meiji University, Higashi-mita 1-1-1, Tama-ku, Kawasaki, Kanagawa 214-8571 Japan

**Keywords:** Benchmarking, Production factors, Reproduction, Sow, Swine

## Abstract

We review critical factors associated with reproductive performance of female breeding pigs, their lifetime performance and herd productivity in commercial herds. The factors include both sow-level and herd-level factors. High risk sow-level groups for decreasing reproductive performance of female pigs are low or high parity, increased outdoor temperature, decreased lactation feed intake, single inseminations, increased lactation length, prolonged weaning-to-first-mating interval, low birth weight or low preweaning growth rate, a few pigs born alive at parity 1, an increased number of stillborn piglets, foster-in or nurse sow practices and low or high age at first-mating. Also, returned female pigs are at risk having a recurrence of returning to estrus, and female pigs around farrowing are more at risk of dying. Herd-level risk groups include female pigs being fed in low efficiency breeding herds, late insemination timing, high within-herd variability in pig flow, limited numbers of farrowing spaces and fluctuating age structure. To maximize the reproductive potential of female pigs, producers are recommended to closely monitor females in these high-risk groups and improve herd management. Additionally, herd management and performance measurements in high-performing herds should be targeted.

## Background

Information technology has enabled the collection and storage of many data about commercial pig herds. As this technology advances there are expanding possibilities for data collection, collaboration and data analysis. Farm data analysis could increase the dissemination of useful information to maximize sows’ reproductive potential, and also improve herd productivity and stable output in breeding herds. However, the use of these farm data is still limited. This review will use farm data to assess critical factors associated with reproductive performance of sows, their lifetime performance and herd productivity in commercial herds, and also discuss the limitations of using commercial herd data for such data analysis.

## Review

### Pigs weaned per sow per year (PWSY)

**Fig. 1:**
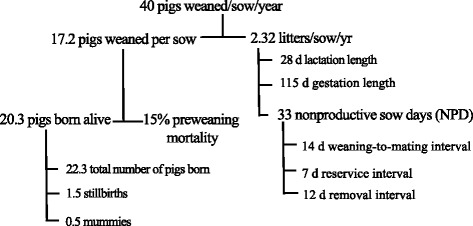
Example of a productivity tree for 40 pigs weaned per sow per year

The number of pigs weaned per sow per year (PWSY) [1] is commonly used as a benchmarking measurement to compare the productivity of breeding herds, either between herds in a country or between countries. The target values for PWSY have increased from 20 to 30 pigs over the last three decades, and it is likely that genetics and sow management can increase PWSY up to 30–40 pigs in the future (Fig. 1). However, even though PWSY is a good measurement for herd productivity in the short term, it is not the best measurement for sow longevity, nor a good measurement for piglet quality or welfare of piglets and sows. There is serious concern that herds with high PWSY may produce many runts or small piglets. The increase in numbers of pigs born alive (PBA), up to 20.3 pigs as shown in Fig. 1, means that the birth weight of piglets is getting lower and also that some light piglets are not able to receive enough colostrum from the sow. This is a problem because lower colostrum intake and lighter birth weights have been associated with a higher preweaning mortality and poorer post weaning growth performance [2]. So piglet quality and welfare may be compromised when sow prolificacy is genetically increased to such a high level, unless genetic improvements are directed to increasing the uterine capacity, the number of functional teats and milk production in sows.

### Reproductive performance in commercial herds

#### Reproductive performance of sows

There are two branches in productivity trees of PWSY in breeding herds (Fig. 1): one is the number of pigs weaned per sow, and the other is the number of litters per sow per year. The number of pigs weaned depends on the number of PBA and preweaning mortality, whereas the number of litters per sow per year depends on non-productive days, lactation length and gestation length.

Sow reproductive performance includes both fertility (e.g.: weaning-to-first-mating interval: WMI) and prolificacy (e.g. PBA). The WMI is highly associated with gonadotropin secretion through the hypothalamic-pituitary-gonadal axis of the sows [3, 4]. In terms of fertility, the number of litters per sow per year is also affected by farrowing rate (FR), as well as by reservice intervals and culling intervals via their effects on non-productive days. Nonproductive days of gilts and sows are also increased by abortion occurrences in commercial herds [5]. Meanwhile, prolificacy measured as PBA, is mainly affected by increasing ovulation rates and decreasing embryonic or fetuses survival rates [6]. However, there appear to be some limitations to genetically increasing PBA due to decreasing piglet quality. In addition, both fertility and prolificacy are influenced by herd effects or the herd’s sow management. Another factor that is critical for sow fertility and prolificacy is sow mortality, because increased mortality increases death intervals and non-productive days, and also decreases PBA which in turn decreases longevity and lifetime productivity in the sows.

#### Lifetime performance

It is important for producers to maximize reproductive potential during sows’ lifetime in order to decrease production costs and economic inefficiency in commercial breeding herds [7]. Lifetime performance includes longevity, which is measured as the number of parity at culling or removal, and also lifetime PBA, lifetime number of pigs weaned and lifetime non-productive sow days [8].

Annualized lifetime PBA is an integrated measurement of sow reproductive productivity that combines lifetime PBA with lifetime sow days. The annualized lifetime PBA is calculated as the number of lifetime PBA divided by a sow’s reproductive herd life days x 365 days. The sow’s reproductive herd life days is the number of days from the date that the sow was first-mated to its removal. Additionally, annualized lifetime pigs weaned can be considered as an integrated measurement of sows’ lifetime reproductive productivity that combines sow performance (i.e. PBA and preweaning mortality) with lactation management including nursing and fostering techniques.

### Sow-level factors for reproductive performance

#### Ordinary factors

##### Low or high parity

Low parity females, especially pregnant gilts and parity 1 sows, have lower reproductive performance than sows in parities 2–5, including lower FR, higher returns and fewer PBA. As the number of parity increases, reproductive performance also increases, reaching a peak between parities 2–5 before it then declines. For example, PBA is greatest between parities 3 and 5, whereas FR is highest between parities 2 and 4. Parity 1 sows also have a prolonged WMI which can be explained by the immature endocrine system in these growing young animals, and also by their low feed intake during lactation which decreases gonadotropin secretion [3] leading to restricted follicle growth in their ovaries.

Additionally, there is a case for a second parity dip which is a decreased PBA in parity 2 sows compared with parity 1 sows [9]. This poor performance in parity 2 sows appears to be associated with low feed intake in the first lactation in parity 1 sows [10]. In general, parity 1 sows in commercial breeding herds commonly do not consume sufficient nutrients and energy in order to grow adequately and reach their mature reproductive performance level.

Aged sows also have lower reproductive performance than parities 2–5 sows. There are various reasons for this lower performance. For example, ovulation and fertilization rates decrease in aged sows. Also, they tend to have increased embryonic mortality or pregnancy loss, and also more stillborn piglets due to slower responses to the space demands by growing fetuses and to the stimuli from parturition processes [11]. Additionally, both aged sows (parity 5 or higher sows) and gilts are at higher risk of having an abortion than parities 2–5 sows [12].

##### High temperature in summer

Fertility and prolificacy decrease during summer months [13]. For example, FR is lowest in summer, and also there are fewer PBA to summer-mated sows than to winter or spring mated-sows. Pigs are short day breeders and so photoperiod is a major factor for reproductive performance of pigs in European countries [14]. The associations between high temperature and reproductive performance have been extensively studied in Asian and European countries [15–18]. It has been hypothesized that reduced reproductive performance in summer occurs through a combination of high temperatures reducing GnRH secretion, and also impairing ovarian follicle development that compromises corpus lutea functions resulting in low progesterone concentrations [13].

Climate data in meteorological stations near studied herds have been used to quantify the association between maximum temperatures and sow performance [15–18]. For example, increased outside temperature decreased FR and total number of pigs born, while it increased returns, WMI and sow mortality.

**Fig. 2:**
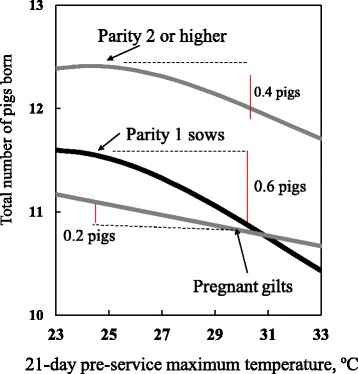
Sensitivity of subsequent total number of pigs born to 21-day pre-service temperature varies with parity [18]. *This study included 27,739 gilts and 127,670 parity records of sows in 95 Japanese herds

Also, various studies have shown that the impact of the outdoor temperature on reproductive performance varies depending on parity number. For example, as the temperature increased from 20 to 30 C^0^, FR in parity 1 sows dropped by at least 10% whereas it only dropped by 2-5% in the other parities [19]. Additionally, as outside temperature increased from 25 to 30 C^0^, the total number of pigs born at subsequent parity decreased by 0.6 pigs for parity 1 sows, whereas it only decreased by 0.2 pigs for parity 0 females (Fig. 2 [18]). Another example is that WMI in parity 1 sows increased by 0.8 days as maximum temperature rose from 25 to 35 °C, whereas in parity 2 or higher sows WMI only increased by 0.3 days [17]. These results indicate that parity 1 sows are 3 times more sensitive to decreases in reproductive performance due to such temperature changes than are gilts or sows at parity 2 or higher. This type of sensitivity in parity 1 sows appears to be related to their immature endocrine system and the low feed intake of parity 1 sows during lactation.

##### Lactation feed intake and its patterns

It is critical to optimize feed intake in lactating sows. Lower lactation feed intake is associated with lower average weaning weight of piglets, prolonged WMI, low FR, as well as more returns or more culled sows due to reproductive failure, and also fewer PBA at subsequent parity [10]. This is particularly the case with parity 1 sows where low feed intake during lactation is detrimental to post weaning reproductive performance. In addition to the amount of feed intake, some lactational feed intake patterns (e.g., major dip) are related to prolonged WMI and more culled sows due to reproductive failure. However, current increases in lactation length and the use of advanced automatic feeders for lactating sows may reduce these risks to reproductive performance.

##### Lactation length

There has been concern about early weaning systems in the U.S.A. being associated with suboptimal reproductive performance, such as prolonged WMI, low FR and fewer PBA at subsequent parity [20]. Also, short lactation length decreases average feed intake during lactation. However, since 2000, the U.S.A. swine industry has been moving from early weaning to increased lactation length [21] in order to improve growth performance in nursery and grower pigs. Also, in the E. U. countries the weaning of piglets from a sow at less than 28 days of age has been prohibited since 2013 [22]. However, there is another concern that some nurse sows with increased lactation length can lose too much of their body reserves due to high milk yields, and so they may have prolonged WMI and lower FR.

##### Number of inseminations or matings

A single insemination with 3 x 10^9^ spermatozoa during the 24 h before ovulation resulted in a fertilization rate of 92–95% on an experimental farm [23]. However, in commercial herds, a single insemination, due in part to late timing, is often related to lower FR and fewer PBA [24, 25]. The occurrence of single inseminations is associated with reserved females, gilt age at first-mating of 150–224 days or 262 or higher days, and WMI of 7 days or more [24]. In the U.S.A. single inseminations have been practiced together with the use of a GnRH antagonist given intravaginally in gel form [26]. This practice may enable the U.S.A. industry to reduce costs while still having reproductive performance levels similar to those with multiple inseminations.

##### Peri-partum period or farrowing event

A farrowing event is a major risk factor for sows in all parities and seasons. Our study showed that approximately 68% sow deaths occurred in the period from 4 weeks before farrowing to 4 weeks after farrowing [27]. The mortality risk for sows increases with parity, with our survival analysis showing that aged sows (e.g., parity 6 or higher) are at the highest risk of dying in the peri-partum period [28].

Sow mortality increases during summer months in the U.S.A. [29]. Also, the summer mortality risk in low parity sows rises with increased outside temperature during the week before the due date. It appears that lower parity females that have immature bodies are more sensitive than multiparous sows to maximum outside temperature around the due date. Pigs are particularly susceptible to heat stress because they have limited sweat glands and a weak cardiovascular system [30]. Heart failure and distortions in abdominal organs are two major causes of death in female pigs [7]. Additionally, some pathways related to postpartum dysgalactia syndrome [31] are possibly associated to sow deaths.

In contrast to higher risks for low parity sows in summer, aged sows are more sensitive to winter minimum outdoor temperature, with more aged sows than low parity females dying around the due date [27]. Such problems may explain the increased aged sow deaths in winter, and be related to aged sow responses to cold or to large variation in daily temperature changes during winter. Therefore, it is recommended that producers pay attention to peripartum pigs in order to perform assisted farrowing, especially when they are likely to experience high or low temperature.

#### Performance factors

Some reproductive performance factors are also predictors associated with other types of performance. So, some performance factors can be used to predict other types of reproductive performance in sows. For example, prolonged WMI is associated with lower FR and fewer PBA, as shown below.

##### Weaning-to-first-mating interval (WMI)

Sows with prolonged WMI have lower FR and fewer PBA than those with WMI 3–6 days [32, 33]. The WMI tends to be increased by short lactation length and low feed intake during lactation [10]. In addition, prolonged WMI is related to a short duration of estrus and to a shorter interval between onset of estrus and ovulation [34, 35]. One consequence of this is an increased risk of inseminating at a suboptimal time, which can be a major cause of low FR and few PBA. As previously mentioned, the use of a GnRH antagonist, given to sows intravaginally, facilitates synchronized estrus in weaned sows. If this practice becomes common, WMI may become a less important factor for other types of reproductive performance.

##### Farrowing failure or return to service

Return-to-service commonly occurs in commercial breeding herds, with approximately 10% of female pigs that fail to farrow being reserved. It has been shown that FR decreases by at least 10% with each reservice [36]. Returned females tend to have estrous behavior that is different from non-returned females. These behavioral differences include having short estrus duration or weak estrus signs, both of which are hard to detect when determining appropriate timing of inseminations.

**Fig. 3:**
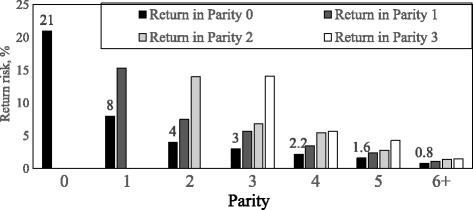
Subsequent return occurrences in first-returned female pigs by parity [37]. *This study contains 65,3528 service records of 114,906 female pigs on 125 EU farms

Analysis of 114,906 females found that 38% had one or more returns in lifetime [37]. Any such occurrence increases non-productive days of female pigs and decreases their productivity. In the study, 33% of the first-returned females had a second return in the same or later parity. In particular, 41% of first-returned gilts had a second return. So females having a return-to-service are at risk for having another return (Fig. 3), and these returned females should be closely monitored.

Reservice intervals account for 30% of NPD, which should be minimized. The reservice intervals are categorized into 3 groups: regular, irregular and late returns with respective re-service intervals of 18 to 24, 25 to 38 and 39 to 150 days post-service [38]. Gilts have more regular returns than sows, and sows have more irregular returns than gilts. A regular return indicates either no conception or failure of maternal recognition. An irregular return implies successful conception but a subsequent early pregnancy loss, and a late return suggests late pregnancy loss [11]. Our study found that 19, 10 and 12% of females that had respective regular, irregular and late returns had a second return of the same type [37].

##### Number of pigs born alive (PBA)

**Fig. 4:**
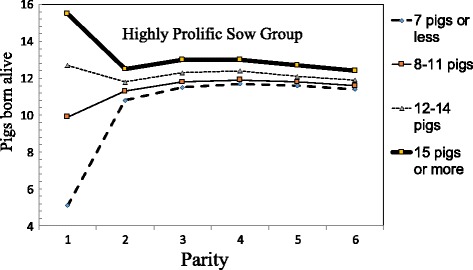
Pigs born alive (PBA) at different parities for 4 sow groups. The 4 groups were categorized by the basis of the 10th, 50th and 90th percentiles of PBA at parity 1 [39]. *This study includes 476,816 parity records of 109,373 sows entered into 125 southern EU herds. Sows were categorized into 4 groups based on the 10th, 50th and 90th percentiles of PBA in parity 1

A southern European study has shown that PBA in parity 1 is a factor that can help producers to identify prolific sows at an early stage [39]. In the study, sows were categorized into 4 groups based on the 10^th^, 50^th^ and 90^th^ percentiles of PBA at parity 1. The sows that had the most PBA in parity 1 continued to produce the most PBA throughout all the subsequent parities (Fig. 4), and also had higher FR up to parity 2. Overall, this most prolific sow group had 23 pigs or more lifetime PBA and 10 pigs or more annualized PBA than the group with the fewest PBA in parity 1. A sow’s PBA is determined by genetic potential and also environmental or management factors [9]. So appropriate management for gilt development is important to increase the numbers of these prolific sows. Also, treating long toes could help to maintain these good sows.

However, no differences have been found between the PBA groups categorized at parity 1 for the number of pigs weaned, WMI or 21-day adjusted litter weights [39, 40]. The lack of any difference between the PBA groups for the number of pigs weaned indicates that extra piglets born to the most prolific sows were fostered on less prolific sows. Also, the lack of any difference in WMI between the PBA groups suggests that the other less prolific groups of sows are not inferior in terms of fertility. Furthermore, the lack of difference in 21-day adjusted litter weights between the PBA groups implies that there were no differences in milk yields between the prolific sows and less prolific sows. So, prolificacy appears to be independent of fertility or milking capability.

##### Birth or weaning weight and preweaning growth rate

Birth or weaning weight and preweaning growth rate are not in the productivity tree of breeding herds in Fig. 1, but they do indicate the quality of piglets, and positively affect their post weaning growth performance. Increased colostrum intake reduces piglet mortality, and increases preweaning and post weaning growth performance [2]. Also, higher preweaning growth in piglets is associated with higher post weaning growth performance [41]. The preweaning growth rate can be increased by management tools such as the use of a milk replacer [42] and two-step nursing [43].

The birth weight and preweaning growth rate of piglets that will become replacement gilts are characteristics of litter-of-origin for subsequent reproductive performance of sows in later life [44]. Higher preweaning growth in replacement gilts is associated with a lower age at puberty. These characteristics appear to affect the subsequent reproductive performance of sows. Lower birth weight is associated with more PBA in the litter, whereas weaning weight and preweaning growth are affected by sow milk production and producers’ lactational management. Therefore, extremely light gilts born to sows that farrowed large PBA should not be selected as replacement gilts. Furthermore, increased preweaning growth is critical to improve subsequent sow performance in later life.

##### Number of pigs weaned

The use of foster-in and nurse sows are common practices because modern sows farrow many PBA per litter, but the practices may impair the metabolic state of sows and decrease post weaning reproductive performance [43, 45]. Sows with an increased number of pigs weaned or having heavier litter weights at weaning could have decreased post weaning reproductive performance due to an increased loss of body reserves and an impaired metabolic state during lactation. However, a study of Danish commercial herds found that nurse sows selected by farm staff tended to be highly prolific sows with a good body condition score and high lactation feed intake [43]. So, nurse sows had more PBA at subsequent parity than non-nurse sows. In addition, it is a widely accepted practice to let parity 1 sows have 13–14 piglets, by using foster-in, in order to develop and stimulate all mammary glands [46]. These sows will have more pigs weaned than other sows.

##### Age of gilts at first-mating

Gilt development and management is critical to optimize the lifetime reproductive performance of sows. However, even though recording the age of gilts at first estrus and the dates of heat-no-serve can help improve gilt development and management, they are rarely recorded in commercial herds in North America. Instead, age of gilts at first-mating is commonly recorded [47], meaning that age of gilts at first-mating is still an important factor in farm data analysis of PBA and lifetime performance in commercial herds. For example, age of gilts at first-mating is associated with type of return. Late returns increase in higher aged gilts [37], which may have degraded ovary and corpora lutea functions, as well as low progesterone concentrations [13]. In contrast, regular returns increase in low aged gilts at first-mating, probably, due to their immature endocrine systems.

Another example of the importance of gilt age at first-mating is that sows first mated at a high age of 278 days or more old, had lower lifetime performance than those mated at an earlier age [39]. This difference is probably because sows with high age at first-mating are likely to become low-efficiency sows as a result of having increased culling intervals due to reproductive failure.

Increased age of gilts at first-mating is also associated with increased PBA in parity 1 [39]. However, this benefit is limited, because even when the gilt age increased from 200 to 300 days there was only a small increase in PBA of about 0.3-0.4 pigs. In the U.S.A., southern European countries and Japan first-mating of gilts is typically carried out at approximately 240 days in order to increase body weights and to ensure more body reserves in replacement gilts that are to be first-mated.

##### Number of stillborn piglets

By definition, stillborn piglets are those piglets that are alive at the initiation of farrowing but die intrapartum [1]. In practice, the stillborn piglets in commercial herds are categorized as piglets found dead behind the sow at the first check up after parturition, with no sign of decomposition [48].

As is the case with age of gilts at first-mating and WMI, the number of stillborn piglets is related to other aspects of reproductive performance. For example, a greater abortion risk for higher parity sows and sows farrowing increased numbers of stillborn piglets has been reported in both southern European and Japanese herds [5, 12]. Such an association could be explained by manual interventions for farrowing difficulties or by infectious agents, such as porcine parvovirus or porcine reproductive and respiratory syndrome virus [11].

### Herd-level factors

Herd characteristics or herd factors, including herd groups by productivity, herd size, management practices, production systems, within-herd variability, age structure and facility types can all be analyzed as herd-level information.

#### High-performing herds

The concept of high-performing herds came from best-practice benchmarking, which has been used to provide target values for reproductive performance and efficiency in breeding herds [49]. Herds can be categorized into two categories based on PWSY: high-performing herds and ordinary herds.

**Fig. 5:**
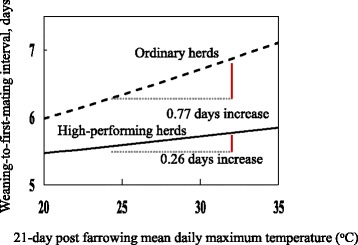
Sensitivity of weaning-to-first-mating interval to post farrowing maximum temperature varies with farm type [17]. *This study comprises 87,428 parity records of 61,558 sows farrowed in 103 Japanese herds, which were classified into high-performing herds and ordinary herds on the basis of pigs weaned per mated female per year

High-performing farms behave differently from ordinary farms. For example, analysis showed that as maximum temperature increased from 25 to 35 C^o^, WMI of sows in high-performing herds increased by only 0.3 days, whereas that in ordinary herds increased by 0.8 days (Fig. 5). The negative effect of high temperature on WMI was 60% less in high-performing herds than that in ordinary herds. So, the results indicate that high-performing herds practice better management that can alleviate 60% of unfavorable effects of high temperature on WMI of sows [17].

Additionally, high-performing herds have higher FR and lower return risks across parity than ordinary herds. Consequently, these high-performing herds have fewer non-productive days, such as reservice interval and culling interval. Also, the high-performing herds have more PBA and more pigs weaned across parities than ordinary herds. In particular, a low FR in low-performing herds is a major contributor to prolonged non-productive days, and so low-performing herds are recommended to improve FR in order to reduce non-productive days [40]. With regards to culling management, high-performing herds have 5-10% lower culling rates from parities 0 to 5 than ordinary herds, but 20 and 200% higher culling rates in parities 6 and 7, respectively [50]. It appears that high-performing herds have a different data management system and culling decision making process [51] to those in low-performing herds.

#### Herd size

In a southern European study, PBA in parity 1 increased by 0.3 pigs as herd size increased from 180 to 1,300 female breeding pigs [39]. Larger herds are associated with higher PWSY due to having fewer non-productive days, shorter farrowing interval and lower preweaning mortality [52], and they may have more rapid genetic improvement and a better production system than small herds. Also, it is possible to hire more specialized staff and use better facilities for large herds than for small herds. Furthermore, high-performing herds have a larger herd size than low-performing herds. So herd size can be used as an indicator of how advanced a production system is, including the amount of investment, the quality of the facilities and human resources, and the level of genetic improvement.

#### Herd management factors

Information relating to herd management factors include gilt development programs, insemination timings, farrowing and lactation management, farrowing spaces and culling guidelines. For example, analysis of insemination timings shows that gilts in herds that perform first insemination immediately after first detection had higher FR than those that delay insemination [53]. Furthermore, when breeding herds were categorized into low FR herds and ordinary herds, based on the 25^th^ percentile of average FR, fewer of the low FR herds inseminated gilts “immediately” or sows by “6-12 h,” compared to ordinary herds [25]. Also, the low FR herds had more single inseminations than ordinary herds, probably because of their later insemination timing. Another finding from herd management analysis is that actual culling intervals for mated gilts and sows were at least 30 days longer than the guideline culling interval [54]. Therefore, it may be advisable for staff on such farms to check the timing of AI in relation to ovulation by using ultrasound scanning of ovaries in gilts [23].

#### Within-herd variability for number of mated females

A consistent flow of pigs through a production facility becomes more important as production systems become more standardized. Within-herd variability in the flow of pigs in a breeding herd can be measured as the number of females mated per week, over a 52-week period. Small within-herd variability in the number of females mated 16–19 weeks previously is associated with higher annual FR, decreased non-productive days, higher PWSY [55] and increased utilization efficiency of farrowing spaces [56]. Additionally, a statistical process control chart [55] is used to monitor within-farm variability in production or pig flow in breeding herds.

#### Number of farrowing spaces

A limited number of farrowing spaces is a frequent pig production bottleneck in most breeding herds. However, more efficient utilization of farrowing spaces can be done by reducing within-herd variability. A survey on commercial swine farms found that a higher utilization efficiency of farrowing spaces is associated with lower within-herd variability measured as the coefficient of variation (%) for the number of females mated 16 weeks previously [56]; the coefficient of variation can be decreased by stabilizing the number of sows and gilts mated, and improving the farrowing rate. Improved farrowing space utilization efficiency enables farms to increase the number of female inventory. Also, decreasing production variation enables producers to solve the bottleneck problem and to produce stable output in commercial herds.

#### Fluctuating age structure

It is necessary to have a stable age structure in breeding herds in order to maintain constant pig production. Using 24-month time-plot charts in parity proportions of parity 0 and parities 3–5 females, 148 herds were categorized into two groups: stable and fluctuating age structure groups [57]. The fluctuating group was illustrated as the plot lines of parity 0 and parities 3–5 proportions crossed over 24 months, whereas the stable group was expressed as the two parity proportion lines never crossed. It was found that there was no difference in average female inventory between the stable age structure herd group and the fluctuating age structure herd group. However, the stable age structure herds had higher FR, lower non-productive days, higher sow longevity and higher PWSY than the fluctuating age structure herds [57]. This is because the herds with a stable age structure had higher proportions of parity 3–5 sows and a lower proportion of gilts than the fluctuating age structure herds. Therefore, age structure variability in breeding herds is associated with lower herd efficiency and sow longevity. Additionally, analysis of the age structure in herds with high efficiency and high sow longevity showed the proportions of parities 0, 1, 2, 3, 4, 5 and 6 females to be 22, 16, 14, 13, 12, 10 and 7%, respectively [50].

#### Boar and semen factors

Farm data analysis of boar semen factors can identify the risk factors for poor performance at semen or boar, sow and herd levels, and it can also determine the motility parameters and the optimum number of motile cells in a dose [58]. However, more research is needed on integrating field data about boar semen factors with reproductive performance of sows.

### Limitations and challenges of data analysis using commercial herd data

There are several limitations with observational studies that would not occur in controlled experiments. For example, herd health, nutrition, management practices and genotypes may not be well controlled in observational studies. Also, some commercial herd data may be recorded incorrectly. Additionally, multiple observations per sow are not independent units of observation. Data in sows within the herd are also in a two-level structure because management practices, production systems, facilities and herd health programs vary between herds: i.e., sows are not independent of the herd. However, even with such limitations, farm data analysis using appropriate exclusion criteria and multi-level statistical models can disseminate practical and readily applicable information to swine veterinarians and producers about production issues that are difficult to investigate by controlled experiments.

## Conclusions

There are sow-level and farm-level risk factors for suboptimal reproductive performance of sows. The sow-level factors include both ordinary factors and performance factors. The ordinary factors include low or high parity, high temperature, decreased lactation feed intake, increased lactation length and a farrowing event, whereas the performance factors are prolonged WMI, returns, few PBA, light birth weight or low preweaning growth rate, foster-in or nurse sow practices, early or late age at first-mating and farrowing stillborn piglets. The herd-level factors include female pigs being fed in breeding herds that have low efficiency, late insemination, high within-herd variability, limited numbers of farrowing spaces, fluctuating age structure and poor semen quality. It is useful for veterinarians to know about the factors affecting sow reproductive performance in order to maximize a sow’s potential and to optimize their client’s breeding herd productivity. However, in order to empower farm data analysis it is necessary to ensure correct data recording, data collection and data integrity checks.

## Abbreviations

FR: Farrowing rate
PBA: The number of pigs born alive
PWSY: The number of pigs weaned per sow per year
WMI: Weaning-to-first-mating interval
